# The kidney histopathological spectrum of patients with kidney injury following snakebite envenomation in India: scoping review of five decades

**DOI:** 10.1186/s12882-024-03508-y

**Published:** 2024-03-21

**Authors:** Priti Meena, Vinant Bhargava, Pallav Gupta, Sandip Panda, Soumyadeep Bhaumik

**Affiliations:** 1 Department of Nephrology, All India Institute Medical Sciences, Bhubaneswar, India; 2 Institute of Renal Science, Sir Gangaram Hospital, New Delhi, India; 3 Department of Pathology, Sir Gangaram Hospital, New Delhi, India; 4 Meta-research and evidence synthesis unit, The George Institute for Global Health, New Delhi, India

**Keywords:** Snakebite envenomation, Acute kidney injury, Kidney histopathology, Acute cortical necrosis, Thrombotic microangiopathy

## Abstract

**Introduction:**

Snakebite is a public health problem leading to about 58,000 deaths every year in India. Kidney injury subsequent to snakebite envenomation is common with a reported prevalence of up to 32%. The current study aims to elucidate the spectrum of kidney histopathology in acute kidney injury (AKI) cases associated with snake bites.

**Methods:**

We searched seven electronic database studies to identify studies describing the histopathological findings in the kidney with snakebite envenomation. Two reviewers independently conducted titles and abstract screening as well as full-text evaluation for the final inclusion decision. Data were extracted as per the standardized form. We conducted narrative synthesis. Studies done exclusively on autopsy findings, in vitro studies, and case reports were excluded.

**Results:**

We retrieved 1464 studies and finally included 28 studies which met the eligibility criteria in the analysis. Most studies were single-centre and the majority were cross-sectional. Overall we included a total of 534 renal biopsies. Russell’s viper bite was the most common cause related to AKI. Acute tubular necrosis was the most common finding followed by acute interstitial nephritis, acute cortical necrosis (ACN), and thrombotic microangiopathy (TMA). Vasculitis changes in vessels were rarely reported. Lesions such as ACN and TMA were associated with poor outcomes.

**Conclusion:**

This analysis supports the notion that renal biopsies are important to guide prognosis and increase our knowledge about post-snake bite AKI pathophysiology.

**Supplementary Information:**

The online version contains supplementary material available at 10.1186/s12882-024-03508-y.

## Summary at a Glance

This article reports an evidence-based synthesis through a scoping review of studies examining kidney histopathology in patients who developed snakebite-induced kidney injuries in India. The study provides insights into aspects of kidney histopathology and pathophysiology for complexities of kidney injury in snakebites.

## Introduction

India has one of the highest burden of snakebite globally with an estimated 58,000 deaths per year from 2000 to 2019 [1]. Half of the global deaths due to snakebite envenomation are from India [2, 3]. Acute kidney injury (AKI) after snakebite envenomation is an important cause of mortality and morbidity. The incidence of AKI after snakebite envenomation as a cause of community-acquired AKI has been reported to be as high as 26% in some Indian studies [4]. Overall snakebite is a significant public health issue in many states of India where its incidence and consequent mortality and morbidity, remain high, although poorly understood [5].

Kidneys are highly vascular organs and therefore are more susceptible to snake venom-induced injury. AKI after snakebite envenomation may be associated with snake venom-induced injury mostly due to hemotoxic or myotoxic snakes of the *Viperidae, Atractaspidae, Elapidae*, and *Colubridae* families [6]. In India *Daboia russelii* and *Echis carinatus* are common snake species known to cause AKI [7].

Patients with snakebite envenomation may clinically present with oliguria, anuria, proteinuria, haematuria, and advanced kidney dysfunction which may require dialysis [6]. Enzymatic toxins found in snake venom contribute to damage across various kidney cell types, affecting glomerular, tubulo-interstitial, and kidney vasculature. While acute tubular necrosis (ATN) is the primary renal pathology, snake envenoming is also significant contributor to acute renal cortical necrosis, often exhibiting varying severity [6]. The aftermath of AKI resulting from a snakebite can lead to diverse outcomes, ranging from death to nonrecovery, partial recovery and complete recovery of renal functions. Despite numerous studies focusing on non-invasive urinary and serum biomarkers for early AKI recognition in high-risk situations, differentiation between prerenal AKI and acute tubular necrosis, and prediction of AKI outcome, there is a notable scarcity of research on the utility of biomarkers in assessing long-term outcomes following AKI. While AKI after snakebite envenomation has been described in the literature, no systematic analysis of the literature on histopathological spectrum and the long-term outcome related to kidney injury in India has been done previously. We aimed to fill this gap by conducting an evidence synthesis of studies documenting renal histopathology findings after snakebite envenomation focusing on its relationship with pathophysiology, clinical findings, and prognosis.

## Methods

We included studies that met the following criteria:

- Type of participants: studies which included humans bitten by a snake (any) in India.
- Concept: studies that report on histopathology (biopsy findings) of the kidney will be included. Studies done exclusively on autopsy findings were excluded.
- Study design - studies were included irrespective of study design, with the exception of in vitro studies, and case reports.
- Restrictions: no restrictions on language or date of publication.

### Data search

We searched electronic databases to identify studies describing the histopathological findings in the kidney with snakebite envenomation from India. We searched seven electronic databases (MEDLINE,EMBASE, CENTRAL, Global Health, PsychINFo, EMCare, SafetyLit) until May 2023 to search for studies and supplemented it with manual screening of references list of included studies. The search strategy was as follows: [(Snakebite) OR (Snakebite envenoming) OR Snake bite induced acute kidney injury)] AND [(renal histopathology) OR (kidney histopathology)) OR ((kidney biopsy) OR (renal biopsy)].

### Article selection

Articles were chosen via a two-step process. At least two independent authors screened each record based on titles and/or abstracts and marked each record as “exclude” or “include ” in a cloud-based artificial intelligence-guided platform (Rayyan **-** https://www.rayyan.ai/). Disagreements at this phase were resolved by consensus. If there was a consensus that an article was unsuitable for inclusion based on the title and/or abstract, it was excluded. Subsequently, two authors conducted an independent screening of the full-text articles and only those that received agreement from both authors were included. In cases where consensus was not initially established, a third author was consulted for discussion until a consensus was achieved. The data was extracted as per a pre-designed data extraction form by two authors and then verified by two other independent authors. We synthesized the data narratively using data as reported in the primary studies, without conducting any additional statistical analysis.

#### Data extraction

Data collection included the study population size, species of snake responsible for AKI, the state in which the study was conducted, the timing of the renal biopsies, the prognosis on long-term follow-up, proportion of survivors, and detailed description of histopathological findings [including light microscopy (LM), Immunofluorescence (IF) and Electron microscopy (EM)] provided in the studies. The renal prognosis of the patients was categorised into 3 categories: (1) persistent renal dysfunction at discharge, (2) progression to end-kidney renal disease and (3) hemodialysis dependency. For the studies providing the long-term follow-up data, follow-up duration was also collected and presented. Only patients with renal biopsy showing signs of TMA such as fibrin thrombi in glomeruli and arterioles were included in the manuscript.

## Results

### Selection of sources of evidence

**Fig. 1:**
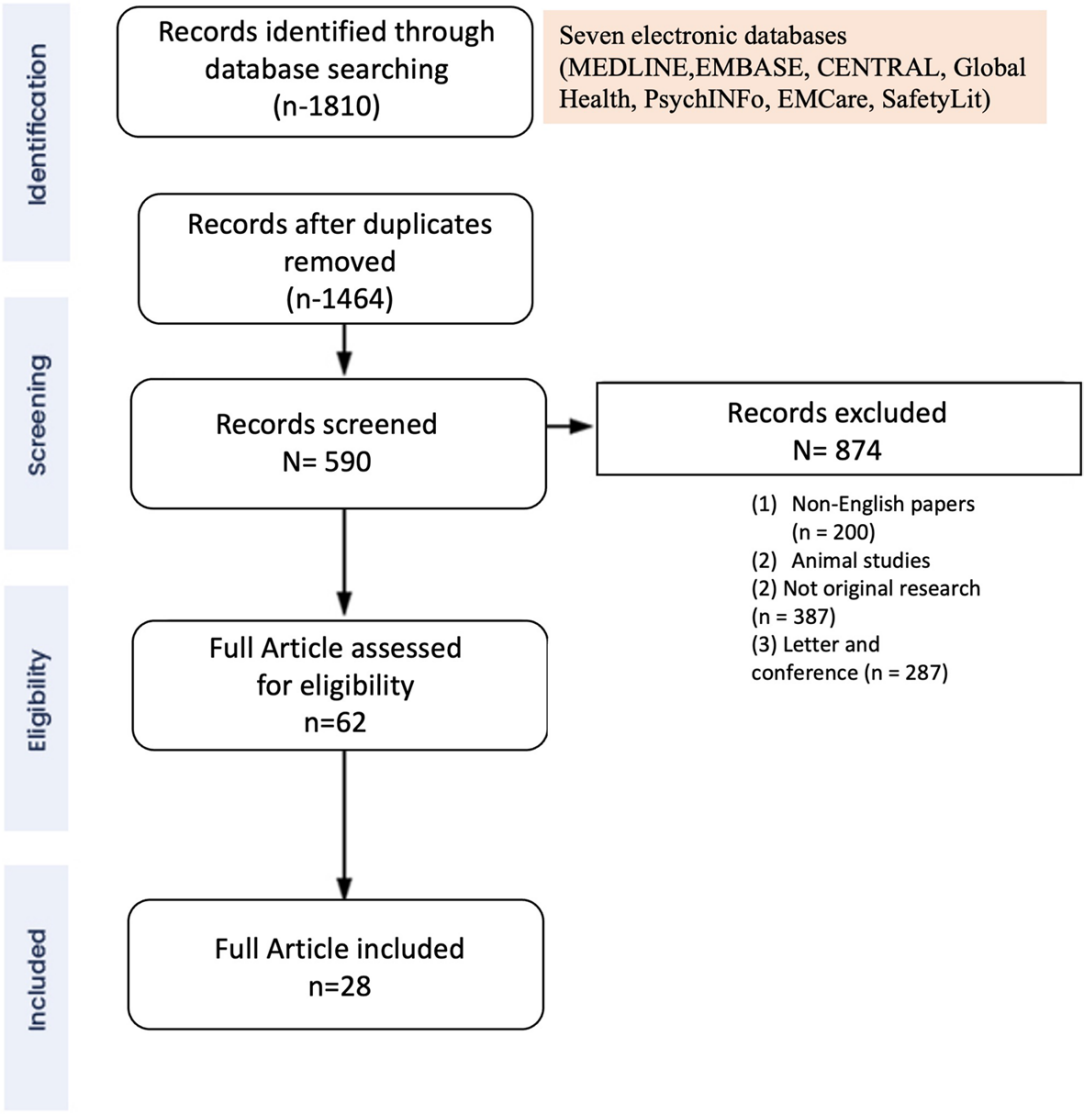
The PRISMA flowchart showing the inclusion of studies

We retrieved 1464 articles (after removing duplicates) but finally included 28 articles which met the inclusion criteria. The PRISMA flowchart showing the inclusion of studies is presented in Fig. 1.

### Characteristics of included studies

The characteristics of the included studies are shown in Table 1 [8–35]. All studies were single-centre with most of them being cross-sectional in nature. Overall we included a total of 534 renal biopsies and 107 renal autopsy findings. Supplementary appendix 1 shows PRISMA-ScR Checklist for the reported studies Two studies, [22, 31] were exclusively done on children with a mean age (± SD) in years of 11.52 ± 2.88 years and 5.8 ± 1.0 years respectively. In the majority of studies, AKI was caused by *Russell’s viper*, however, in some studies, *Echis carinatus* and sea snakes were also identified to be associated with kidney involvement [15–17, 27, 29]. 

**Table 1:** Characteristics of included studie**s**

	First Author	Year of publication	Population	Study design	State	Exclusively done on children	Snake species	Indication/ timing of biopsy	Total Number of Snakebite Patients	Total Number of snake bite induced AKI cases	Total Number of Renal Biopsies
1	Chugh et al., [8]	1975	AKI following snake-bite	Cohort with follow-up	Chandigarh	No	*Russell’s viper*	NR	69	8	8
2	Basu et al., [9]	1977	Patients with AKI following *viperine* bite	Cross-sectional	West Bengal	No	*Russell’s viper*	Early diuretic phase	NR	45	37
3	Shastry et al., [10]	1977	Patients with AKI following snake bite	Cohort with follow-up	Tamilnadu	No	*Russell’s viper*	Day 4 to 31	NR	19	19
4	Sarangi et al., [11]	1980	AKI following *viperine* snake bite	Cross-sectional	Odisha	No	*Russell’s viper*	After oliguric phase of AKI	48	23	22
5	Date et al., [12]	1982	Patient with AKI following snake bite	Cross-sectional	Tamil nadu	No	*Russell’s viper*	Biopsy timing varied from 5–22 days.	NR	9	9
6	Chugh et al., [13]	1984	AKI following snake bite. Study was conducted to describe renal histopathological lesions in AKI following snakebite in humans and to know the effects of *viperine* venoms on the renal structure and function in subhuman primates.	Cross-sectional	Chandigarh	No	NR	During the polyuric phase.	157	45	35
7	Date et al., [14]	1986	Patient with AKI following snake bite	Cross-sectional	Tamilnadu	No	*Russell’s viper*	NR	NR	24	15
8	Acharya et al. [15],	1989	AKI following *viper-ine* snake bite	Prospective	Maharashtra	4 patients were children below the age of 10years.	*Russell’s viper* and *Echis carinutus* were the main snakes identified. One case of renal failure with sea snake bite was reported	1.5 to 8 weeks	NR	50	29
9	Chugh et al., [16]	1989	All patients with snakebite with treated for *viper* bite [presumed] poisoning.	Cross-sectional	Chandigarh	No	*Russell’s viper* and *Echis carinatus*	NR	246	70	44
10	BV Mittal et al., [17]	1994	Patient with AKI following snake bite were included	Cross-sectional	Maharashtra	No	*Russell’s viper* in 14 (34%), *Echis carinatus* or saw scaled viper in 20 (48%) and sea snake in one case.	Varied from 3–8 weeks	253	41	41
11	Chugh et al., [18]	1994	Patients dialysed for AKI and diagnosed to have ACN	Cohort with follow-up	Chandigarh	No	NR	NR	16	16	16
12	Vijeth et al., [19]	1997	Adult cases of viper bite with systemic envenomation	Observational	Pondicherry	No	*Russell’s viper*	Persistant renal dysfunction	40	13	3
13	Golay et al., [20]	2012	Patients with AKI after snake bite	Cohort with follow-up	West Bengal	No	*Russell’s viper*	In patients who remained oliguric or the serum creatinine did not decrease to less than 50% of the attained peak value at the end of 3 weeks	NR	42	13
14	Waikhom et al. [21],	2012	Post snake bite patients who developed dialysis-requiring AKI and had survived	Prospective observational	West bengal	No	*Russell’s viper*	NR	499	410	10
15	Waikhom et al., [22]	2013	All pediatric patients with AKI following Russell’s viper bite	Prospective study, supplemented by a retrospective chart review	West bengal	Children < 15 years were included	*Russell’s viper*	NR	NR	61	5
16	Golay et al. [23],	2013	Post snake bite patients with AKI who had survived	Prospective study,	West bengal	No	*Russell’s viper* in 7 out of 9 cases (77%), 2 were unidentified	In whom AKI did not resolve by the end of 3 weeks	126	-	4
17	Mukhopadhyay et al., [24]	2016	All snakebite patient who received hemodialysis	Cross-sectional	West bengal	29 patients were of age ≤ 18 years	NR	NR	460	203	3
18	Vikrant et al., [25]	2017	Patients with definitive history of snake bite; clinical picture consistent with snake bite, as presence of fang marks or cellulitis or coagulopathy or neuroparalysis; presence of AKI as defined using KDIGO criteria based on serum creatinine and presence of at least one or more indication of RRT	Retrospective	Himachal pradesh	No	NR	Patients who remain oligoanuric or whose serum creatinine did not decrease satisfactorily at the end of 3 weeks underwent kidney biopsy	447	81	22
19	Priyamvada et al., [26]	2016	Patients diagnosed with snake envenomation-induced AIN	Retrospective	Puducherry	No	*Daboia russelii* 22.2% (*n* = 41) *Echis carinatus*, 14.1% (*n* = 26). The species of snake was not identified in 117 patients (63.6%).	Renal biopsies are performed if the serum creatinine remains greater than 2 mg/dL 4 weeks post-envenomation.	NR	88	7
20	Dinesh kumar et al., [27]	2018	Patients with AKI after snake bite and kidney biopsy showing AIN	Prospective observational	Tamil Nadu	No	*Russell’s viper* 25% (*n* = 5), *saw-scaled viper* 20% (*n* = 4), and *krait* 5 % (*n* = 1)	Renal biopsy were performed if kidney dusfunction persisted for more than 3 weeks or earlier if persistant anuria for more than 2–3 weeks or pathology other than ATN was suspected for example fragmented RBCs in peripheral smear.10–40 days	196	196	Total 85 biopsies were done. Results for Twenty (23.5%) patients who underwent biopsy had AIN were presented in the study
21	Shaktirajan et al., [28]	2018	Cases of AKI with renal biopsy showing pigment nephropathy	Retrospective observational	Tamil Nadu	No	NR	Patients with persistent oliguria for > 7 days and renal failure for > 14 days despite supportive treatment.	10	10	10
22	Priyamvada et al., [29]	2020	All adult patients with AKI following haemotoxic snake envenomation were recruited	Prospective observational	Puducherry	No	Species identification was not done in 2 patients, and the rest were *Daboia russelii* (as reported by the patients)	Persistant renal dysfunction beyond 3 months	420	184	3
23	Rao et al., [30]	2019	Patients with age > 18 years with definitive history of snake bite( consistent clinical picture like the presence of fang marks, cellulitis, coagulopathy, neuroparalysis) and presence of AKI (as per KDIGO 2012 guidelines)	Case-record‐based retrospective analysis	Karnataka	No	NR	At day 18 in one patient and at day 31 in another patient	103	103	2
24	Islam et al. [31]	2020	Paients with hematotoxic snake envenomation characterized mainly by a positive 20 min whole blood clotting test (WBCT) admitted in pediatric emergency ward	Comparative	West Bengal	Yes ( Mean age 5.8 ± 1) years	NR	Children suffering who suffered from permanent renal damage and who died.	371	139	64
25	Kumar M et al., [32]	2022	Patients with AKI with (1) Definitive history of snake bite; (2) Clinical picture suggestive of snake bite with the presence of a fang mark.	Retrospective recruitment followed by a prospective follow	Chennai	No	Snake species was identified in 29 patients (18.2%)—*Cobra* in three, Russell’s viper in 14, *Saw scaled viper* in seven and *Krai*t in five.	NR	769	159	41
26	Ariga et al. [33]	2021	All adult patients with AKI (as per KDIGO criterion) survived the episode and were discharged	Retrospective and prospective observational (Ambidirectional)	Puducherry	No	Unidentified 98 (50.7%) *Russell’s viper* 85 (445) *Saw-scaled viper* 10 (5.3%)	NR	NR	193	6
27	Acharaya et al., [34]	2023	All adult patients with AKI (as per KDIGO criterion) following haemotoxic snake envenomation were recruited	Prospective	Odisha	no	*Viperidae* species	NR	NR	202	30
28	Prema et al., [35]	2023	Patients with hemoglobulin cast nephropathy	Retrospective analysis	NR	NR	NR	NR	NR	NR	16

*NR *Not reported or not clear, *HD *Hemodialysis, *ACN *Acute cortical necrosis

### Timing and indication of renal biopsy

In the majority of studies, kidney biopsies were done after 2–3 weeks from the onset of AKI, The most common indication for renal biopsies was persistent renal dysfunction or dialysis dependency. Pathological changes varied depending upon the time lag from the onset of AKI to kidney biopsy. Histopathological findings in Kidney biopsies of included studies are shown in Table 2. Kidney tissue obtained during the early diuretic phase of acute tubular necrosis (ATN) revealed epithelial degeneration in tubules, tubular vacuolation, desquamation, and severe intertubular interstitial oedema with regenerative changes developing at the later stages of AKI (after 3 to 8 weeks) [9, 30, 31]. Interstitial haemorrhage was more common in the 1st week of the bite [15]. The nature of the tubular casts also changed with the time lag between the bite and kidney biopsy. Hyaline casts with degenerating cellular-granular casts were more commonly observed in earlier stages whereas red blood cells (RBC) casts appeared later.

**Table 2:** Kidney biopsy findings chart

SL No	Primary Author	Light microscopy			IF	EM
		Glomerular changes	Tubulointerstitial changes	Vascular changes		
**1**	**Chugh et al. 1975**, [8]	ACN in *n* = 3 (50%) of the cases. Surviving glomeruli in areas of necrosis showed fibrin thrombi and some proliferation of mesangial cells.	ATN in *n* = 3 (50%) of the cases	Not mentioned specifically	NR	NR
**2**	**Basu et al., 1977**, [9]	ACN in 2 (5.4%) of the cases. Glomerular tuft necrosis *n* = 5 (13.4%),Isolated Glomerular thrombosis *n* = 7 (19%)	ATN in 32 (86.4%) cases. Peritubular infiltration of inflammatory cells *n* = 6 (16.2%) Medullary haemorrhagen = 14 (27%)	Vessel wall necrosis *n* = 5 (13.4%) Fibrin thrombi in arteriole *n* = 2 (5.4%)	NR	NR
**3**	**Shastry et al., 1977** [10]	ACN in 3 (15.7%) of the cases. Focal mesangial hypercellularity in *n* = 6 (32%).	ATN in 12 (63.2%) of cases, Interstitial haemorrhage *n* = 3 (15.7%). Presence of hyaline, granular and heme cast. Vacuolation and regeneration. Intertitial fibrosis in 1 case Interstitial oedema (10.5%) Interstitial inflammation *n* = 10 (52.6%)	Not mentioned specifically	NR	NR
**4**	**Sarangi et al., 1980**, [11]	ACN in one case. Aneurysmal capillary dilatation and inflammatory cell infiltration were seen in glomerulus	Acute tubular injury *n* = 3 (13.6%), Acute tubular necrosis *n* = 6 (27.2%), Presence of interstitial haemorrhage oedema (5 cases) and RBC tubular cast.	Not mentioned specifically	NR	NR
**5**	**Date et al., 1982**, [12]	Glomeruli was normal except for focal mesangial expansion and focal prominence of parietal epithelium. One case had necrosis of cortical tissue	ATN *n* = 9 ( 100%) .The interstitium was expanded and infiltrated by mononuclear cells and scattered eosinophils predominantly at the cortico-medullary junction.	No abnormality	NR	Glomeruli: Swollen bowman’s capsule epithelium containing numerous organelles. The visceral epithelium revealed microvilli, patchy foot process fusion and intracytoplasmic lipid vacuoles. Glomerular capillaries had irregular thickening and wrinkling of glomerular basement membrane. Mesangial expansion was present in all biospies. Subendothelial electron dense deposits were seen in one case and one case had features of ACN. Blood vessels: Swollen endothelial cells containing numerous vacuoles and a dilated smooth endoplasmic reticulum. Interstitium: Oedematus intertubular tissue and infiltration of inflammatory cells including eosinophils, 6mast cells, plasma cells and lymphocytes and some macrophages and basophils.
**6**	**Chugh et al., 1984**, [13]	ACN *n* = 10 (28.5%). Superficial cortical glomeruli were more severely congested compared to deep juxtamedullary glomeruli. Hilar glomerular capillaries were partly filled with recent thrombus.	ATN *n* = 23 (65.7%). PCT showed shrunken, pyknotic nuclei or no stainable nuclei with cytoplasmic cloudy swelling and considerable tubulorrhexis. Distal convolutions were collapsed. Collecting ducts contained brown heme casts or pigments.	The large intrarenal arteries were deeply congested. The intertubular capillaries were crowded with neutrophils. In some cases venules and veins were congested with inflammatory cells.	NR	NR
**7**	**Date et al., 1986** [14]	ACN *n* = 3 ( 20%)	ATN *n* = 12 (80%)	NR	Not done	Done in 7 (46.6%) biopsies. Biopsies revealed platelet and fibrin clusters in glomeruli and small calibre vessels
**8**	**Acharya et al., 1989** [15]	ACN in 7 (24.1) cases. Ballooning of glomerular capillaries (*n* = 10), swollen endothelial cells (*n* = 10), splitting of basement membrane (*n* = 19). Mesangial cell proliferation (*n* = 5). Proliferative glomerular changes in 5 cases.	ATN in 10 (40% ) cases with evidence of tubular degeneration in 17% cases (42.5%)	Vasculitis like changes in vesselsN = 3 (7%) cases. Fibrin thrombi in capillaries, *n* = 6(24.2% )cases.	Not done	Not done
**9**	**Chugh et al., 1989** [16]	Patchy ACN in 4 cases (9%) and diffuse cortical necrosis in 8 patients (18%).	ATN was present in 32 (73%) of cases, AIN in 3 patients (6.8%).	Necrotizing arteritis of the interlobular arteries along with thrombophlebitis of the arcuate vein and its tributaries in 2 cases.	Dense deposits of C3 were seen in the walls of afferent and efferent arterioles in cases of necrotizing arteritis.	Not done
10	Mittal et al., 1994 [17]	ACN in 25 (60.9%) cases. Focal proliferation of mesangial cell *n* = 5 (12.1%). Mild mesangial proliferation *n* = 22 (53.6%). Mesangiolysis *n* = 7 (17.7%) Ballooning of glomerular capillaries *n* = 13 (31.7%), swollen endothelial cells *n* = 16 (39%), splitting of basement membrane *n* = 11 (26.8%). Capillary thrombi *n* = 5 (12.1%).	Tubular necrosis *n* = 5 (12.1%), Tubular regeneration *n* = 2 (4.8%). Pyelonephritis complicating tubular necrosis and one autopsied case revealed abscesses. Haemorrhagic interstitial nephritis with haemorrhage in subcapsular area in case.	Other than in cases of ACN. Not mentioned specifically	Done in 7 cases [17] IgG (weak +):2 cases IgM (weak +):1 case C3 (+):2 cases in mesangial area. Presence of fibrin in mesangial area: 1 case	Not done
11	Chugh et al., 1994 [18]	ACN in all 16 patients	NR	NR	NR	NR
12	Vijeth et al. [19]	NR	ATN in 3 (100%) cases	NR	NR	NR
**13**	**Golay et al., 2012** [20]	Normal glomeruli	ATN in 8 (21.5% ) and AIN in 5 (11.9%) cases. Extensive interstitial inflammation was observed in all the AIN cases, with pre-dominantly lymphocytic infiltration, in one case where eosinophils were predominant.	Normal Blood vessels	No IF deposits	NR
**14**	**Waikhom et al., 2012** [21]	ACN in 3 patients (30%)	ATN in 6 patients (60%). AIN in1 case	Not specifically mentioned	NR	NR
**15**	**Waikhom et al., 2013** [22]	ACN in one case.	ATN in 3 patients.(60%).AIN in1 case	Not specifically mentioned	NR	NR
**16**	**Golay et al., 2013** [23]	ACN in one (25% ) case	ATN in 3 (75% ) patients	Not specifically mentioned	NR	NR
**17**	**Mukhopadhyay et al., 2016** [24]	ACN in one out of 3 patients	ATN in 2 out of 3 patients. (66.6%)	NR	NR	NR
**18**	**Vikrant et al., 2017** [25]	Patchy ACN in one case.	ATN in 20 (91%) cases. In 9 (40.9%) ATN was associated with mild to moderate AIN. One (4.5%) patient only had moderate AIN	Not specifically mentioned	Findings not specifically mentioned	NR
**19**	**Priyamvada et al., 2016** [26]	Diffuse mesangial proliferation; in 1(20% )out of 5 case. Rest were normal	ATN in 4 cases (57.1%). AIN in 5 out of 7 cases (71.4%). Lymphocyte-predominant infiltration in all patients. 4 patients (57.1%). had admixture of other cell types including eosinophils, neutrophils, and plasma cells. Neutrophil cast in was seen 1 patient.	Not specifically mentioned	Weak 1 + C1q deposits in mesangium of 1 patient	NR
**20**	**Dinesh kumar et al., 2018** [27]	Not specifically mentioned	AIN was reported in 20 ( 23.5%) out of 85 biopsies. Marked infiltration of eosinophils and lymphocytes was seen along with tubular injury.	Not specifically mentioned	Negative in all patients	NR
**21**	**Shaktirajan et al., 2018** [28]	None of the patients had significant glomerulosclerosis or any other specific glomerular abnormality	Snake bite envenomation was present in 10 out of 46 patient of pigment induced nephropathy. All renal biopsies revealed ATN with pigment casts in the tubules. No interstitial fibrosis or tubular atrophy were noted	Not specifically mentioned	NR	NR
**22**	**Priyamvada et al. 2020** [29]	1 out of 2 patients had features of chronic TMA	Not specifically mentioned	Not specifically mentioned	NR	NR
**23**	**Rao et al., 2019** [30]	Biospsy of both patient was suggestive of ACN with fibrin thrombi in glomerular capillary lumen and arterioles. TMA was present in both cases.	One biopsy showed evidence of AIN along with TMA	Fibrin thrombi was present in in glomerular capillary lumen and arterioles.	NR	NR
**24**	**Islam et al., 2020** [31]	ACN in *n* = 8 (12.5%) cases.	ATN in *n* = 25 (39.1) %. cases	NR	NR	NR
**25**	**Kumar M et al., 2022** [32]	• 5 (12% ) out of 41 had ACN. • 4 (9.7 )out of 41 had TMA • One patient had TMA with renal cortical necrosis	• ATN in *n* = 18 (44%) cases. • *N* = 5 (12% ) biopsies showed pigmented casts • AIN was reported in 9 ( 22%). • *N* = 5 (12% )cases had ATN with AIN	NR	NR	NR
**26**	**Ariga et al., 2021** [33]	NR	Out of 6, *n* = 2 (50%) were ATN and *n* = 4 (66.6% ) were AIN	NR	NR	NR
**27**	**Acharya et al., 2023** [34]	• ACN in *n* = 7 (23%)	• AIN was reported in *n* = 6 ( 22%). • ATN in *n* = 18 (59.6)% cases.	NR	NR	NR
**28**	**Prema et al.** [35]	NR	• All 16 snake bite patients had ATN alongside hemoglobulin cast nephropathy. • Hemoglobin casts were seen present in the PCT. The casts were bright red to brown on hematoxylin and eosin stain weakly PAS positive, argyrophilic on Jones methenamine silver stain, and granular to globular in texture. Hemoglobin IHC was positive on the pigment casts. Two patients showed myoglobin immunostain positivity.	NR	NR	NR

*NR *Not reported, *HD *Hemodialysis, *ACN *Acute cortical necrosis, ATN Acute tubular necrosis, AIN Acute interstitial nephritis, TMA Thrombotic microangiopathy, PCT Proximal convoluted tubules, PAS Periodic acid-Schiff, *IHC *Immunohistochemistry

### Renal Biopsy findings

#### Glomerular lesions

Changes in the glomerulus were largely underreported. Glomerular changes occurring with acute cortical necrosis (ACN) were the most commonly reported finding. However, thrombotic microangiopathy (TMA) and focal and diffuse mesangial proliferation have also been reported [30, 32]. Studies also reported necrosis of glomerular tuft and isolated glomerular thrombosis [9]. Specific glomerular changes like ballooning and dilatation in the glomerular capillary loops, focal proliferation of mesangial cells, endothelial cell swelling, splitting of glomerular capillary basement membrane were reported by Mittal et al. and Acharya et al. [15, 17].

#### Tubulointerstitial changes

ATN was the most frequently encountered finding in kidney biopsy tissue. The incidence varied from 30 to 100% [8, 9, 11, 12, 14–16, 18, 23, 25–27, 32, 34]. Hyaline, granular, or pigment casts were frequently seen along with dilated tubules lined with flattened epithelium and desquamation of necrotic cells [12, 28]. Acute interstitial nephritis (AIN) has been reported non uniformly [25–27, 32, 34]. In a series by Priyamvada et al., 5.7% of snakebite-induced AKI were reported to have AIN on kidney biopsy [26]. The kidney biopsy demonstrated mixed infiltrate of predominantly lymphocytes and variable proportions of other cells like neutrophils, eosinophils, and occasional plasma cells. Neutrophil cast was reported in one patient. The prevelance of AIN was slightly higher in other series; out of 85 biopsies, 20 (23.5%) patients had AIN [27]. Marked infiltration of eosinophils and lymphocytes with substantial tubular injury was reported. Golay et al. observed extensive interstitial inflammation, with a predominant lymphocytic infiltration [20]). In viperine envenoming, “hemorrhagic interstitial nephritis” characterized by haemorrhages in the interstitium with tubular necrosis and RBC congestion in the tubular lumen was reported [10, 17]. One study reported features of pyelonephritis complicating ATN [17]. Patchy and diffuse areas of hemorrhagic necrosis in the cortex and widespread medullary areas have also been reported in in Russell viper’s envenomation [9]. 

##### Pigment induced nephropathy

Sakthirajan et al., in their analysis of pigment-induced nephropathy, found snake envenomation as the most frequent etiology of rhabdomyolysis [28]. Out of the 26 patients with rhabdomyolysis, 10 were caused by snakebite envenomation. All biopsies revealed features of acute tubular injury and pigment casts. In an another series, among a cohort of 56 patients diagnosed with hemoglobin cast nephropathy, the second most prevalent etiology, following drug-induced cases, was attributed to snake envenomation-induced hemolysis. This particular cause was observed in 16 patients, accounting for approximately 28.4% of all patients. Positive myoglobin immunostaining was observed in two patients who had suffered snake bites envenomation [35]. 

### Vascular changes

Vasculitis-like changes including necrotizing arteritis, thrombophlebitis, and vessel wall necrosis have been described in *Russell’s viper* bite cases [9, 13, 15]. Severe congestion of large intrarenal vessels along with venules and veins and crowding of neutrophils in intertubular capillaries were reported by Chugh et al. [16].

### Thrombotic microangiopathy

Rao et al. described a series of TMA in AKI induced by snake-bite envenomation [30]. In this study, out of 103 patients post snake-bite envenomation AKI, 19 (18.5%) had clinical features of TMA. However, renal biopsy was done in only 2 patients which showed features of TMA such as fibrin thrombi in glomerular capillary lumen and arterioles with patchy cortical necrosis. Priyamvada et al. also reported chronic TMA in patients who developed CKD following snakebite envenomation [29]. 

### Acute cortical necrosis (ACN)

Following ATN, ACN was the second most common finding reported in patients with snake-bite AKI. Its incidence varies between 5 and 100% of biopsies reporting it [10–13, 15–18, 21, 22, 24, 31, 32, 34]. Fibrin and platelet thrombi were found predominantly in lobar and sublobar arteries. In an analysis by Chugh et al. including 113 cases of ACN, viperine snake-bite envenomation was one of the major causes responsible for 16 (14.2% ) of total ACN cases [18]. 

### Immunofluorescence (IF)

Only a few studies reported IF findings [13, 17, 20, 26, 27]. A study showed dense C3 deposits in the afferent and efferent arteriolar walls in cases of necrotizing arteritis [16]. Mittal et al. demonstrated IF results in 7 patients and reported a weak positivity for IgG and IgM, along with C3 positivity in the mesangial area [17]. Priyamvada et al. found a weak C1q deposition in the mesangium [26]. Other studies failed to demonstrate any deposits in IF [20, 27]. 

### Electron microscopy

Studies describing electron microscopic findings were scanty [12, 14]. Date et al. provided a detailed description of ultrastructural findings. The authors reported swollen cytoplasm of bowman’s capsule epithelium with visceral epithelium showing blebs, microvilli, patchy foot process fusion, and intracytoplasmic lipid vacuoles. The basement membrane of the glomerular capillaries was thick and wrinkled. In blood vessels, endothelial cells were swollen and cytoplasmic protrusions were seen to be protruding into the lumen. Infiltration of inflammatory cells was found in the interstitium. Intracytoplasmic bodies were seen in the proximal tubules representing degenerating organelles.

### Renal biopsy findings and outcomes

The kidney outcomes in AKI following snakebite envenomation varied from partial or complete recovery of kidney functions to progression to end-stage kidney disease (ESKD) resulting in dialysis dependence. Studies reported that snakebite envenoming patients who did not recover their kidney functions had diffuse cortical necrosis and TMA as the predominant pathological findings. Patients with acute tubular injury were reported to respond well to conservative management and dialysis contrary to those with ACN who only responded partially or not at all [10]. Supplementary Table 1 shows renal and patient outcomes in biopsied patients. Sarangi et al. demonstrated that the clinical presentation and prognosis of the patients were directly proportional to the severity of renal histopathological lesion on the kidney biopsy [11]. Lower survival rates were reported in ACN. 8 out of 10 patients (80%) who had bilateral renal cortical necrosis, and 4 out of 23 patients with less severe acute tubular lesions died (*P* < .001). Other series reported a mortality rate of up to 100% in patients with ACN [8]. 

Studies also reported that as compared to non-TMA cases of AKI, TMA cases were associated with more advanced azotemia at presentation with an almost universal requirement for dialysis. These patients required a longer duration of renal replacement therapy (RRT), and hospitalization and had higher chances of progressing into chronic kidney disease (CKD) with higher mortality insinuating a poor prognosis [29, 30]. Golay et al. reported worse clinical outcomes while comparing cases with and without AIN [23]. 

## Discussion

To the best of our knowledge, this is the first systematic synthesis of evidence presenting an analysis of renal histopathology findings after snakebite envenomation in India. ATN followed by ACN were the common renal histopathological lesions reported in multiple studies but TMA, mesangial proliferation, pigment-induced nephropathy and AIN were also reported. Globally other histopathological findings such as proliferative glomerulonephritis after *Echinatus carinatus* bite and crescentic and diffuse proliferative glomerulonephritis after *Russell’s viper* have also been reported [36–38]. None of the studies in our analysis reported such changes. Studies included in our review show that snake-bite AKI patients present with oliguria, hematuria, and advanced azotemia requiring dialysis, with persistent oliguria lasting for more than 2–3 weeks implicating the occurrence of ACN [10, 13, 18]. .

**Fig. 2:**
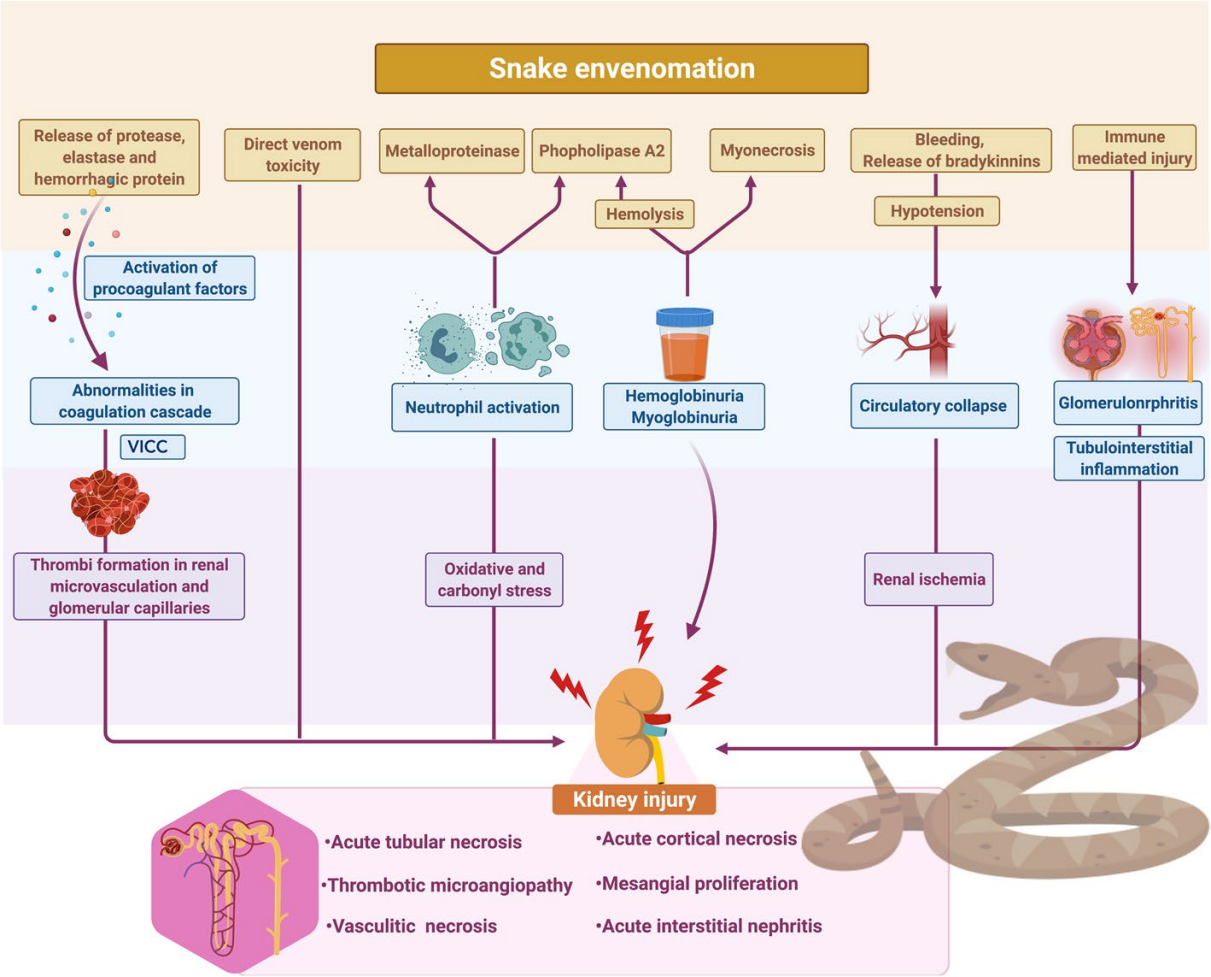
Proposed mechanism of snake bite-induced kidney injury VICC: Venom-induced consumption coagulopathy

The mechanism of kidney injury in snakebite envenomation is usually multifactorial, it is an interplay of various cytokines, vasoactive substances like endothelin, and other immune mediators [39–41]. It can be attributed to numerous reasons such as direct nephrotoxicity of the venom, circulatory collapse, intravascular hemolysis with hemoglobinuria, extensive myonecrosis causing myoglobinuria, or Venom-Induced Coagulopathy (VICC). Various proteases, amino acid esterase enzymes, and hemorrhagic proteins present in viperine snake venom can activate procoagulant factors and induce coagulation cascade abnormalities including bleeding diathesis and VICC [39]. Postulated mechanism in snake bite associated AKI is shown in Fig. 2 mechanism of snake.

The pathogenesis of VICC distinguishes itself from Disseminated Intravascular Coagulation (DIC). While DIC arises from multifaceted mechanisms culminating in fibrin deposition, VICC’s coagulation activation originates primarily from a snake procoagulant toxin, not the tissue factor/factor VIIa pathway implicated in DIC [42]. VICC’s severity varies based on the toxin’s action within the coagulation pathway, ranging from mild fibrinogen consumption to severe deficiencies in fibrinogen, factor V, and factor VIII. Crucially, VICC lacks evident fibrin deposition, microvascular thrombotic obstruction, and resulting organ damage, in contrast to DIC [43]. VICC predominantly manifests as bleeding, with the risk determined by whether the snake toxin acts as a hemorrhaging agent, such as by metalloproteinase prothrombin activators. These activators not only trigger the coagulopathy pathway but also induce vascular injury, heightening the risk of bleeding, a distinctive feature absent in DIC where vessel walls remain unaffected [40]. Pigment-induced nephropathy was seen in the cases complicated with intravascular hemolysis leading to hemoglobinuria. It was particularly more common in viper and crotalid snakebites whereas sea snakes were categorically myotoxic [39]. 

The infiltration of various cells such as lymphocytes, monocytes, eosinophils, basophils, and mast cells in the interstitium in cases of ATN was hypothesized to be either a consequence of some immunologically mediated reaction to antigens released from necrotic renal tubules or due to homocytotropic antibody-mediated reaction [44, 45]. However, their activation and accumulation in the interstitium have also been argued to be a similar phenomenon as seen in delayed hypersensitivity reactions [46]. It was postulated that lesions occurring in the early phase could be the consequence of the direct toxic effect induced by snake venom and the lesions developing late could be plausibly immunologically mediated resulting in the formation of fibrin thrombi similar to those in hemolytic uremic syndrome (HUS) and VICC [14]. 

Vasculotoxic effects and complement activation by the snake venom probably via alternate pathways (based on the C3 without immunoglobulin deposition on the arterial wall) are plausible mechanisms for the pathogenesis of these vascular lesions [17, 26]. 

Kidney biopsies during the initial week are usually not feasible and pose a high risk due to coagulation abnormalities and thrombocytopenia. This is a deterrent in establishing a correlation between structural and functional findings in the kidneys. Kidney biopsy remains an essential tool to predict prognosis. ACN and TMA lesions impart worse prognosis and usually progress to ESKD and dialysis-dependent stages in most cases. The majority of studies have not adequately differentiated between outcomes associated with TMA versus non-TMA cases. Notably, the challenge of performing biopsies in TMA cases is compounded by factors such as advanced azotemia, low platelet count, and anaemia. Furthermore, TMA-like changes in blood vessels have been identified concomitantly with other lesions like ACN and ATN contributing to an overall portrayal of poor prognosis in these lesions. This inherent difficulty in obtaining biopsies and the overlapping nature of TMA with other renal conditions, collectively lead to a generalization of unfavourable outcomes in the existing literature, Additionally, studies like those conducted by Priyamvada et al. raise concerns about potential high selection bias, particularly in patients proceeding to renal biopsy. Mostly the patients with persistent renal dysfunction and having poor renal outcomes undergo renal biopsy. This bias may compromise the ability to conduct a comprehensive comparative outcome analysis between different types of renal histopathological lesions. The role of steroids and plasmapheresis in the management of AIN and snake envenomation-induced TMA respectively is yet to be elucidated.

Our review also indicated some gaps in evidence, which need to be investigated in the future. Most studies on the domain are cross-sectional in nature providing no insights on prognosis and other issues. As such information on the interpretation of pathophysiology, and exploration of treatments for kidney management is not possible. Interpretations around the utility of doing renal biopsies in the early phases of AKI in informing clinical practice are also not possible from cross-sectional studies. Another key challenge is the lack of uniformity in reporting the cause of AKI by biopsy. Different histopathologic changes may have been attributed to the same disease process, redundancy in the nomenclature cannot be ruled out. There is a need for consensus standardisation in the domain.The absence of species identification and its consequential effect on the interpretation of the review’s findings is another limitation.

The venom compositions of snakes differ both within and across species. This will lead to varying causes of AKI and, thus, potentially varied histological findings.

## Conclusion

In conclusion, this scoping review represents the inaugural systematic synthesis of evidence examining renal histopathology findings in snakebite envenomation-induced kidney injury in India over five decades. ATN and ACN were predominant histopathological lesions, with additional reports of TMA, mesangial proliferation, and AIN. Prognostically, ACN lesions signal poorer outcomes, often progressing to ESKD. Gaps in evidence, including limited longitudinal studies and the lack of standardized reporting, necessitate future research to enhance our understanding and inform clinical practice in managing snakebite-induced kidney injury.

### Supplementary Information

**Additional file 1.**

**Additional file 2: Supplementary Table 1. **Showing renal and patient outcomes in biopsied patients.

## Data Availability

The datasets used and/or analysed during the current study available from the corresponding author on reasonable request.

## Abbreviations

ATN: Acute tubular necrosis
AIN: Acute interstitial nephritis
ACN: Acute cortical necrosis
AKI: Acute kidney injury
CKD: Chronic kidney disease
EM: Electron microscopy
ESKD: End-stage kidney disease
IF: Immunofluorescence
KDIGO: Kidney Disease:Improving Global Outcomes
LM: Light microscopy
HD: Haemodialysis
RRT: Renal replacement therapy
TMA: Thrombotic microangiopathy
VICC: Venom-induced consumption coagulopathy
