# Supplementary material for: The kidney histopathological spectrum of patients with kidney injury following snakebite envenomation in India: scoping review of five decades
Source: BMC Nephrol. 2024 Mar 21;25:112. doi: 10.1186/s12882-024-03508-y (PMC10958888; doi:10.1186/s12882-024-03508-y)
Supplement: Supplementary file 2 — Additional file 2: Supplementary Table 1. Showing renal and patient outcomes in biopsied patients. [file 12882_2024_3508_MOESM2_ESM.docx]

| **Supplementary Table 1 showing renal and patient outcomes in biopsied patients.** | First Author | Total Number of Snakebite Patients | Total Number of snake bite induced AKI cases | Total Number of Renal Biopsies | Number of patient with persistent renal dysfunction | Number of patients who progressed to ESKD | Requirement of dialysis  among biopsied patients | Died (n) |
| --- | --- | --- | --- | --- | --- | --- | --- | --- |
| 1 | Chugh et al, (8) | 69 | 8 | 8 | None | None | 6 | 3 |
| 2 | Basu et al, (9) | NR | 45 | 37 | NR | NR | 31 | 16 |
| 3 | Shastry et al, (10) | NR | 19 | 19 | All patients with ACN | NR | 15 | 1 |
| 4 | Sarangi et al, (11) | 48 | 23 | 22 | None | None | 1 | 1 patient with ACN |
| 5 | Date et al, (12) | NR | 9 | 9 | None | None | 8 | None |
| 6 | Chugh et al, (13) | 157 | 45 | 35 | NR | NR | NR | 8 patients with bilateral renal cortical necrosis and 4 patients with ATN died |
| 7 | Date et al, (14) | NR | 24 | 15 | All 3 patients of ACN | NR | 22 | 1 |
| 8 | Acharya et al,(15) | NR | 50 | 29 | NR | NR | 45 | 20 |
| 9 | Chugh et al, (16) | 246 | 70 | 44 | NR | NR | NR | NR |
| 10 | BV Mittal et al, (17) | 253 | 41 | 41 | NR | NR | NR | 6 out of 10 ACN cases died |
| 11 | Chugh et al, (18) | 16 | 16 | 16 | NR | NR | NA | NR |
| 12 | Vijeth et al, (19) | 40 | 13 | 3 | NR | NR | NR |  |
| 13 | Golay et al, (20) | NR | 42 | 13 | 5 | 1 | All 5 AIN patients | None |
| 14 | Waikhom et al,(21) | 499 | 410 | 10 | 10 | NR | 10 | NR |
| 15 | Waikhom et al, (22) | NR | 61 | 5 | NR | NR | NR | NR |
| 16 | Golay et al,(23) | 126 | - | 4 | 2 | NR | NR | NR |
| 17 | Mukhopadhyay et al, (24) | 460 | 203 | 3 | NA | NA | 3 | NR |
| 18 | Vikrant et al, (25) | 447 | 81 | 22 | NR | NR | NR | NR |
| 19 | Priyamvada et al, (26) | NR | 88 | 7 | One patient had creatinine of 1.6 at 5th month of follow up | 1 patient remained HD dependant at 3 months of follow up. | 5 | None |
| 20 | Dinesh kumar et al, (27) | 196 | 196 | 20 | During the median follow up of 24 ± 6 months  6 patients developed CKD | NR | 20 | None |
| 21 | Shaktirajan et al, (28) | 10 | 10 | 10 | NR | NR | NR | NR |
| 22 | Priyamvada et al, (29) | 420 | 184 | 3 | During a median follow-up duration of 15.5 months all 3 biopsied patient had persistent renal dysfunction | 1 | 1 | None |
| 23 | Rao et al, (30) | 103 | 103 | 2 | None | 1 | 2 | None |
| 24 | Islam et al (31) | 371 | 139 | 64 | 48 | NR | NR | 16 |
| 25 | Kumar M et al, (32) | 769 | 159 | 41 | 11 | 1 | 5 |  |
| 26 | Ariga et al (33) | NR | 193 | 6 | 3 | 1 | 1 | 0 |
| 27 | Acharaya et al, (34) | NR | 202 | 30 | 3 | 3 | 3 | 16 |
| 28 | Prema et al, (35) | NR | NR | 16 | NR | NR | NR | NR |
